# Simultaneous Determination of Atorvastatin and Aspirin in Human Plasma by LC–MS/MS: Its Pharmacokinetic Application

**DOI:** 10.3797/scipharm.1206-12

**Published:** 2012-08-06

**Authors:** Ramakrishna Gajula, Nageswara Rao Pilli, Vasu Babu Ravi, Rambabu Maddela, Jaswanth Kumar Inamadugu, Srinivasa Rao Polagani, Sobha Busa

**Affiliations:** Wellquest Clinical Research, Mirrakamshetty Mall, Ramanthapur, Hyderabad 500013, India.

**Keywords:** Atorvastatin, Aspirin, Acetylsalicylic acid, Liquid–liquid extraction, Chromatography, Pharmacokinetics

## Abstract

A simple, rapid, and sensitive liquid chromatography tandem mass spectro-metric (LC–MS/MS) assay method has been developed and fully validated for the simultaneous quantification of atorvastatin and aspirin in human plasma using a polarity switch. Proguanil and furosemide were used as the internal standards for the quantification of atorvastatin and aspirin, respectively. The analytes were extracted from human plasma by the liquid–liquid extraction technique using methyl *tert*-butyl ether. The reconstituted samples were chromatographed on a Zorbax XDB Phenyl column by using a mixture of 0.2% acetic acid buffer, methanol, and acetonitrile (20:16:64, v/v) as the mobile phase at a flow rate of 0.8 mL/min. Prior to detection, atorvastatin and aspirin were ionized using an ESI source in the multiple reaction monitoring (MRM) mode. The ions were monitored at the positive *m*/*z* 559.2→440.0 transition for atorvastatin and the negative *m*/*z* 179.0→136.6 transition for aspirin. The calibration curve obtained was linear (*r^2^* ≥ 0.99) over the concentration range of 0.20–151 ng/mL for atorvastatin and 15.0–3000 ng/mL for aspirin. The method validation was performed as per FDA guidelines and the results met the acceptance criteria. A run time of 3.0 min for each sample made it possible to analyze more than 300 human plasma samples per day. The proposed method was found to be applicable to clinical studies.

## Introduction

Hyperlipedemia is the elevation in the bloodstream of lipids including fats, fatty acids, cholesterol, cholesterol esters, phospholipids, and triglycerides. The control of hyper-lipedemia is important for the prevention of atherosclerosis and its associated conditions such as coronary heart disease, ischemic cerebrovascular disease, peripheral vascular disease, ischemic cerebrovascular disease, and peripheral vascular disease [1, 2]. The 3-hydroxy-3-methylglutaryl coenzyme A (HMG–CoA) reductase inhibitors (statins) are the most commonly used drugs in the treatment of hyperlipidemia. Statins competitively inhibit the enzyme HMG–CoA reductase, which is involved in the rate–limiting step of cholesterol biosynthesis in the liver, resulting in the up–regulation of the low–density lipoprotein (LDL) receptor and the lowering of LDL cholesterol in the blood [3, 4].

Atorvastatin (CAS no. 134523-00-5) is a potent and competitive inhibitor of the enzyme HMG–CoA reductase, the rate–limiting enzyme in cholesterol biosynthesis in the liver. Atorvastatin induces a significant reduction in total cholesterol (TC), LDL–C, and plasma triglycerides (TG) [5, 6]. In addition, atorvastatin exerts an antithrombotic effect in patients with type 1 diabetes and dyslipidemia [7]. Aspirin (CAS no. 98201-60-6), also known as acetylsalicylic acid, is one of the most widely prescribed antipyretic, analgesic, and anti–inflammatory agents [8]. At low doses (<100 mg), aspirin is employed as an antithrombotic agent to selectively inhibit cyclooxygenase–dependent platelet aggregation [9].

Aspirin in combination with atorvastatin decreases the risk of major adverse cardiac events via suppression of the inflammatory response [10]. Several studies observed the potential lipid-modifying effects of the combination therapy of statins with aspirin [10, 11].

Many LC–MS/MS methods have been reported for the determination of atorvastatin [12–22] individually or in combination with other drugs in biological samples. The major disadvantages of these methods include less sensitivity [13, 20, 21], requiring a larger sample volume [12, 13, 17, 19], longer chromatographic run time [12, 13, 15, 17, 18], and a narrow linearity range [13, 17, 19]. Similarly, numerous LC–MS/MS methods are described in the literature to determine the amount of aspirin in different biological fluids [23–25]. Of the methods applied to the analysis of aspirin, either the chromatographic run time was long [23, 24] and the plasma volume high [23], or the method was too insensitive for routine application [23, 25]. Some methods [12, 14, 18, 19, 22], which can be applied for the quantitation of one drug in biological fluids selectively, cannot be applied satisfactorily for the simultaneous determination of atorvastatin and aspirin. For pharmaco-kinetic and bioequivalence studies of atorvastatin in combination with aspirin, it is recommended to perform the quantitation of atorvastatin and aspirin simultaneously. To date, no LC–MS/MS method has been reported for the simultaneous determination of atorvastatin and aspirin in human plasma.

In view of the above, the authors have attempted to develop an LC–MS/MS method for the simultaneous determination of atorvastatin and aspirin in human plasma. A combination tablet formulation containing atorvastatin, 10 mg, and aspirin, 75 mg, (Stator ASP, Piramal Health Care Ltd, Mumbai, India) is commercially available in the Indian market for the management of hyperlipidemia and atherosclerosis-related conditions. The authors feel that this method will help to analyze these two drugs in formulations that are now available in the market as well as in plasma samples. The present paper describes a simple, selective, and sensitive method, which employs the liquid–liquid extraction technique for sample preparation, and liquid chromatography with electropspray ionisation–tandem mass spectrometry for the simultaneous quantitation of atorvastatin and aspirin in human plasma. The method did not show any interference from commonly used drugs and was successfully applied to a pharmacokinetic study of atorvastatin and aspirin in healthy male volunteers. The authenticity in the measurement of the study data was demonstrated through incurred sample reanalysis.

## Experimental

### Materials and reagents

**Fig. 1.**
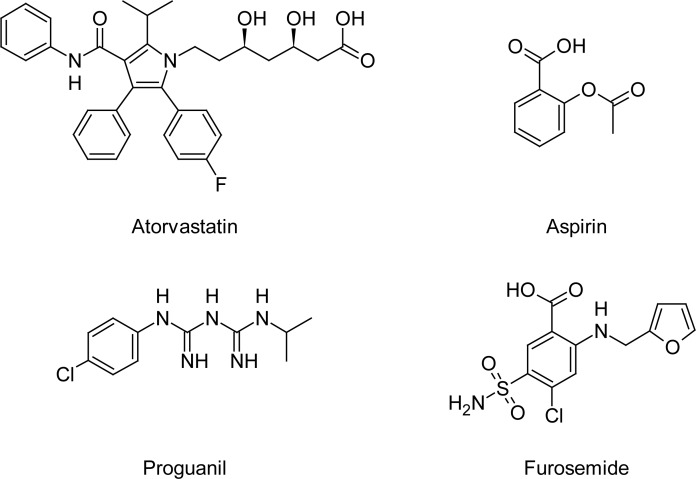
Chemical structures of atorvastatin, aspirin, proguanil, and furosemide.

The reference sample of aspirin (100.0% pure) was purchased from LGC Promochem, India, and that of atorvastatin (97.90%) from Neucon Pharma Ltd, India. The pure samples of furosemide (99.22%) and proguanil hydrochloride (99.60%) used as internal standards in this study were obtained from Hetero Drugs Limited, India. The chemical structures of these compounds are presented in Figure 1. The water used for the LC–MS/MS analysis was purified with the Milli-Q water purification system procured from Millipore (Bangalore, India). HPLC grade acetonitrile, methanol, and methyl *tert*-butyl ether (MTBE) were purchased from J.T Baker (Phillipsburg, USA). Analytical grade acetic acid and formic acid were purchased from Merck (Mumbai, India). The control human plasma sample was procured from Cauvery Diagnostics and Blood Bank (Secunderabad, India).

### Chromatographic conditions

An HPLC system (Shimadzu, Kyoto, Japan) consisting of a Zorbax XDB Phenyl column (75 × 4.6 mm, 3.5 μm; Agilent Technologies, Santa Clara, CA, USA), a binary LC–20AD prominence pump, an auto sampler (SIL–HTc), and a solvent degasser (DGU–20A_3_) were used for the study. Aliquots of the processed samples (20 μL) were injected into the column, which was kept at 40 ºC. An isocratic mobile phase of a mixture of 0.2% acetic acid buffer, methanol, and acetonitrile (20:16:64, v/v) was delivered at a rate of 0.8 mL/min into the electrospray ionization chamber of the mass spectrometer.

### Mass spectrometer conditions

Quantitation was achieved with MS–MS detection using a MDS Sciex API–4000 mass spectrometer (Foster City, CA, USA) equipped with a Turboionspray™ interface. The MS/MS method consisted of two periods containing both a negative and positive ionization mode. Specifically, the mass spectrometer operated in the negative detection mode for about 1.1 min for aspirin, followed by a period of 1.9 min in the positive mode for atorvastatin. The total run time was set at 3.0 min. The main working parameters of the mass spectrometer are summarized in Table 1. The analytical data obtained were processed by Analyst software™ (version 1.4.2).

### Preparation of plasma standards and quality controls

Stock solutions (1.0 mg/mL) of atorvastatin, proguanil, and the furosemide were prepared in methanol, whereas that of aspirin was prepared in 0.2% acetic acid in acetonitrile. From these stock solutions, appropriate dilutions were made using a 50:50, v/v mixture of acetonitrile and water as a diluent to produce working standard solutions of 0.01, 0.02, 0.04, 0.20, 0.40, 0.81, 2.02, 3.24, 4.84, 6.04 μg/mL for atorvastatin and 0.60, 1.20, 3.01, 6.00, 12.0, 24.0, 48.1, 72.0, 96.0, 120.0 μg/mL for aspirin. Stock solutions of atorvastatin, aspirin, proguanil, and furosemide were found to be stable for 30 days at 2–8 °C (data not shown). The calibration curve (CC) standard solutions of atorvastatin and aspirin in blank plasma (containing 70 μL aliquot of 150 mg/mL potassium fluoride in 1 mL of plasma) were prepared by spiking them with the appropriate volumes of their working solutions (25 μL of atorvastatin and 25 μL aspirin working solution), giving the final concentrations of 0.20, 0.40, 1.01, 5.06, 10.1, 20.2, 50.6, 81.0, 121, and 151 ng/mL for atorvastatin, and 15.0, 30.1, 75.2, 150, 301, 601, 1202, 1800, 2400, and 3000 ng/mL for aspirin. The CC samples were analyzed along with the quality control (QC) samples for each batch of plasma samples. The QC samples were prepared at five different concentration levels of 0.20 (lower limit of quantitation, LLOQ), 0.61 (low quality control, LQC), 22.3 (middle quality control, MQC–1), 75.4 (MQC–2), and 130 (high quality control, HQC) ng/mL for atorvastatin and 15.8 (LLOQ), 45.0 (LQC), 450 (MQC–1), 1500 (MQC–2), and 2500 (HQC) ng/mL for aspirin in blank plasma. All of the prepared plasma samples were stored at −70 ± 10 °C.

### Sample processing

An aliquot of 250 µL of the human plasma sample was mixed with 25 μL of the internal standard working solution (1000 ng/mL of combined dilution of proguanil and furosemide). To this, 50 μL of the potassium dihydrogen phosphate buffer (1M) was added. After vortexing for 15 s, a 4 mL aliquot of the MTBE was added using a Dispensette Organic pipette (GmbH, Wertheim, Germany). The sample was shaken for 10 min using a reciprocating shaker (Scigenics Biotech, Chennai, India) and then centrifuged for 4 min at 4000 rpm using a Heraeus Megafuse 3SR centrifuge (Japan). The organic layer (3.0 mL) was transferred into a 10 mL glass test tube and evaporated at 40ºC under a stream of nitrogen. The dried extract was reconstituted in 500 μl of the mobile phase and transferred into autoinjector vials. From these, a 20 μl aliquot was injected into the chromatographic system.

### Method validation

The validation of the above method was carried out as per US FDA guidelines [26]. The parameters determined were selectivity, specificity, matrix effect, linearity, precision, accuracy, recovery, stability, and dilution integrity. Selectivity was assessed by comparing the chromatograms of six different batches of blank plasma obtained from six different sources including one lipemic and one hemolyzed plasma. The potential interference from acetaminophen, diphenhydramine, pantoprazole, nicotine, ibuprofen, caffeine, and pseudoephedrine was evaluated. Sensitivity was determined by analyzing six replicates of plasma samples spiked with the lowest level of the calibration curve concentrations. The matrix effect was checked with six different lots of K_2_ EDTA plasma. Three replicate samples each of LQC and HQC were prepared from different lots of plasma (36 QC samples in total). For checking the linearity, standard calibration curves containing at least 10 points (non–zero standards) were plotted (0.20–151 ng/mL for atorvastatin and 15.0–3000 ng/mL for aspirin). In addition, blank plasma samples were also analyzed to confirm the absence of direct interferences. Intra–day precision and accuracy were determined by analyzing six replicates at five different QC levels on two different days. Inter–day precision and accuracy were determined by analyzing six replicates at five different QC levels of five different runs. The recoveries of atorvastatin, aspirin, furosemide, and proguanil were determined by comparing the peak area of the extracted analyte standard with the peak area of the non–extracted standard. Recoveries of atorvastatin and aspirin were determined at a concentration of 0.61, 45.0 (LQC), 75.4, 1500 (MQC–2), and 130, 2500 (HQC) ng/mL, respectively, whereas the internal standards were determined at a concentration of 1000 ng/mL. The dilution integrity was performed to extend the upper concentration limit with acceptable precision and accuracy. Six replicates each at a concentration of about 1.6 times the uppermost calibration standard were diluted 2– and 4–fold with blank plasma. The diluted samples were processed and analyzed.

Stability tests were conducted to evaluate the analyte stability in stock solutions and in plasma samples under different conditions. The stock solution stability at room temperature and refrigerated conditions (2–8 °C) was performed by comparing the area response of the analytes (stability samples) with the response of the sample prepared from the fresh stock solution. Benchtop stability (8 h), processed sample stability (autosampler stability for 48 h, wet extract stability for 24 h, and reinjection stability for 24 h), freeze–thaw stability (4 cycles), and long–term stability (50 days) tests were performed at the LQC and HQC levels using six replicates at each level. Samples were considered to be stable if assay values were within the acceptable limits of accuracy (85–115%) and precision within ≤15% RSD.

### Pharmacokinetic study design

A pharmacokinetic study was performed in healthy male subjects (*n* = 6). The ethics committee approved the protocol and the volunteers provided their informed written consent. Blood samples were collected following the oral administration of atorvastatin (20 mg film-coated tablet) and aspirin (75 mg) at the pre–dose, and 0.083, 0.167, 0.25, 0.33, 0.417, 0.5, 0.67, 0.83, 1, 1.25, 1.5, 1.75, 2, 2.5, 3, 3.5, 4, 6, 8, 12, 16, and 24 h, in K_2_ EDTA vacutainer collection tubes (BD, Franklin, NJ, USA) containing a 70 μL aliquot of 150 mg/mL potassium fluoride (to minimize the hydrolysis of aspirin to salicylic acid in the blood). The tubes were centrifuged at 3200 rpm for 10 min and the plasma was collected. Immediately after collection, the plasma samples were subjected to flash–freezing and stored at −70 ± 10°C until their use. The plasma samples were spiked with the IS and processed as per the extraction procedure described earlier. Along with the clinical samples, the QC samples at low, middle 1, middle 2, and high concentration levels were also assayed in triplicate. The plasma concentration–time profile of atorvastatin and aspirin was analyzed by the non–compartmental method using WinNonlin Version 5.1.

## Results and discussion

### Method development

The mass parameters were tuned in both the positive and negative ionization mode for the analytes. Good response was found in the positive ionization mode for atorvastatin and the negative ionization mode for aspirin. A negative–to–positive ionization switch mode was used to detect the two analytes in order to achieve the best sensitivity for aspirin and atorvastatin. Data in the MRM mode were considered, which showed better selectivity. The positive ion spray mass spectrum revealed a protonated molecule by monitoring the transition pairs of the *m/z* 559.2 precursor ion to the *m/z* 440.0 for atorvastatin and the *m/z* 254.2 precursor ion to the *m/z* 170.1 product ion for the proguanil. The negative ion spray mass spectrum revealed a deprotonated molecule by monitoring the transition pairs of the *m/z* 179.0 precursor ion to the *m/z* 136.6 for aspirin and the *m/z* 329.2 precursor ion to the *m/z* 285.0 product ion for the furosemide. As earlier publications have discussed the details of the fragmentation patterns of atorvastatin [19], aspirin [27], proguanil [28], and furosemide [29], we are not presenting the data pertaining to this.

Chromatographic conditions, especially the composition of the mobile phase, column type, flow rate, and column oven temperature were optimized through several trials to achieve high resolution and an increased intensity of the signals of the analytes, as well as the short run time. The presence of a small amount of acetic acid in the mobile phase improved the detection of the analytes. It was found that a mixture of the isocratic mobile phase consisting of 0.2% acetic acid, methanol, and acetonitrile (20:16:64, v/v) could achieve this purpose, and was finally adopted as the mobile phase. The Zorbax XDB Phenyl (75 mm x 4.6 mm, 3.5 μm) column produced a good peak shape and response, even at the lowest concentration level for both of the analytes. The mobile phase was operated at a flow rate of 0.8 mL/min. As the selection of the column oven temperature is important for proper resolution between the negative and positive ionization modes, it was set at 40 °C. The retention times of aspirin, furosemide, atorvastatin, and proguanil (0.94, 0.96, 1.33, and 2.06 min, respectively) were low enough, allowing a short run time of 3.0 min.

The liquid–liquid extraction (LLE) technique was employed for the sample preparation in this work. LLE is helpful in producing a spectroscopically clean sample, and in avoiding the introduction of non–volatile materials onto the column and MS system, and also minimizing the experimental cost. Clean samples are essential for minimizing ion suppression and the matrix effect in LC–MS/MS. Among the different solvents checked alone and in combination for their suitability, MTBE was found to be optimal, because it produced a clean chromatogram for the blank sample and yielded the highest recovery for the analytes from the plasma.

A good internal standard must mimic the analyte during extraction and compensate for any analyte on the column. For LC–MS/MS analysis, the use of stable isotope–labeled drugs as internal standards proves to be helpful when a significant matrix effect is possible. Isotope–labeled analyte was not available to serve as the IS, so in the initial stages of this work, several compounds were investigated to find a suitable IS, and finally proguanil was found to be the best for the quantification of atorvastatin in the positive ionization mode, and furosemide for aspirin in the negative ionization mode.

### Selectivity and chromatography

**Fig. 2.**
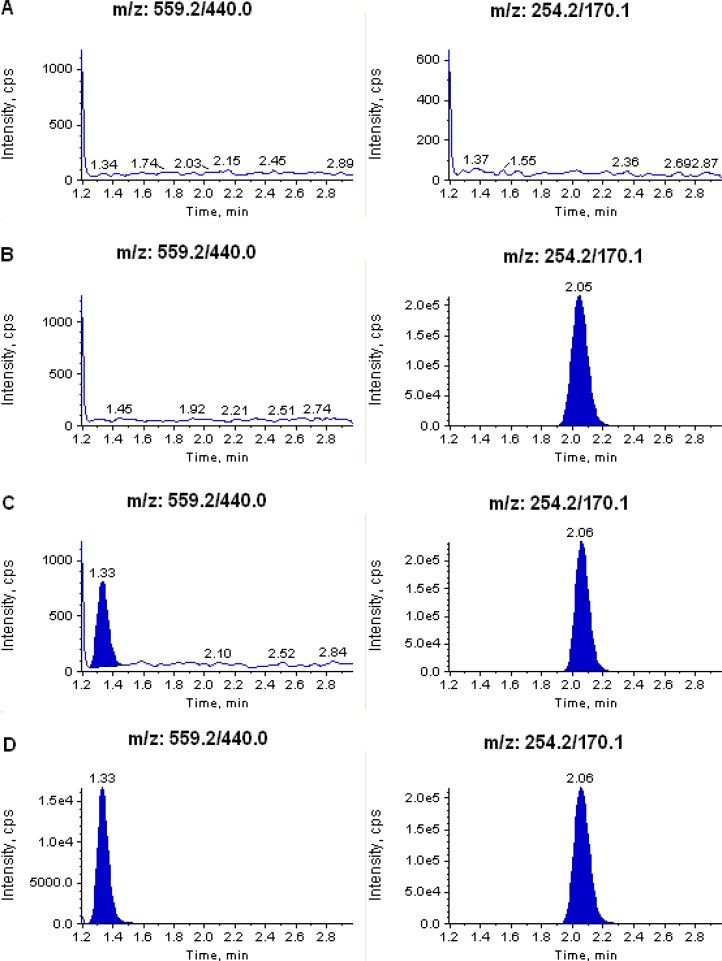
Typical MRM chromatograms of atorvastatin (left panel) and IS (right panel) in (A) human blank plasma; (B) human plasma spiked with IS; (C) an LLOQ sample along with IS; and (D) a 1.5 h plasma sample (10.1 ng/mL) showing atorvastatin peak along with IS obtained following oral administration of 20 mg of atorvastatin tablet to a healthy volunteer.

**Fig. 3.**
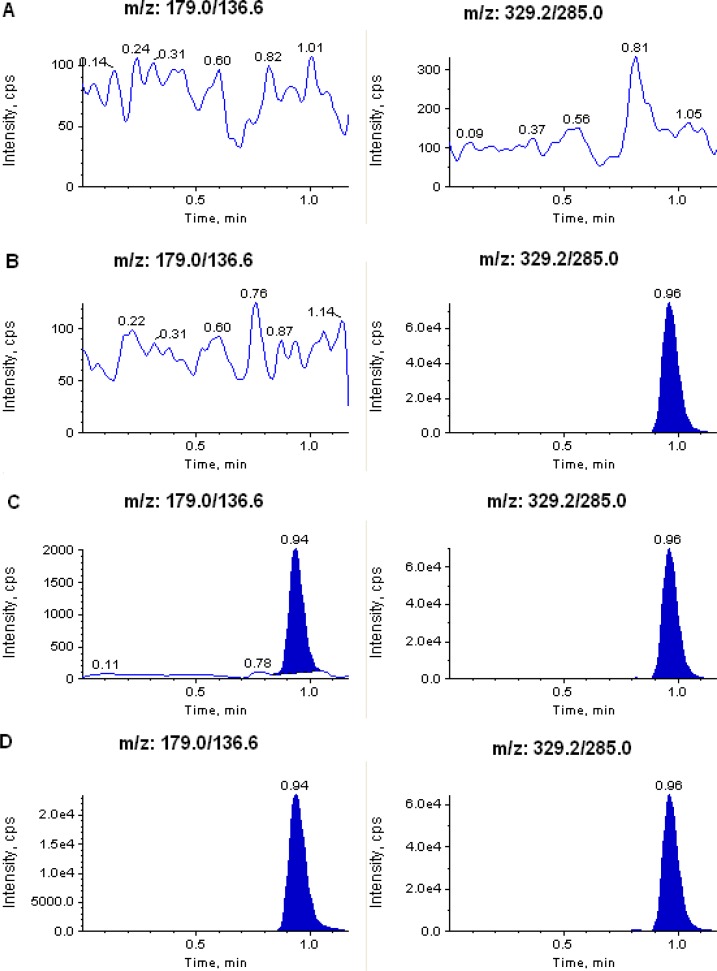
Typical MRM chromatograms of aspirin (left panel) and IS (right panel) in (A) human blank plasma; (B) human plasma spiked with IS; (C) an LLOQ sample along with IS; and (D) a 1.5 h plasma sample (304 ng/mL) showing aspirin peak along with IS obtained following oral administration of 75 mg of aspirin tablet to a healthy volunteer.

The degree of interference by endogenous plasma constituents with the analytes and the internal standards was assessed by the inspection of chromatograms derived from processed blank plasma samples. As shown in Figure 2 and 3, no significant direct interference in the blank plasma traces was observed from endogenous substances in drug–free plasma at the retention time of the analytes and the internal standards. Similarly, no interference was observed from commonly used medications such as acetaminophen, diphenhydramine, pantoprazole, nicotine, ibuprofen, caffeine, and pseudoephedrine.

### Sensitivity

The lowest limit of reliable quantification for the analytes was set at the concentration of the LLOQ. The precision and accuracy at the LLOQ concentration were found to be 4.81% and 101%, and 3.79% and 96.5% for atorvastatin and aspirin, respectively. We also found that in the presence of the atorvastatin HQC concentration, no interference found was at the retention time of aspirin at the LLOQ. Similarly, the sensitivity of atorvastatin was not affected by the HQC concentration of aspirin (data not shown).

### Matrix effect

No significant matrix effect was observed in all of the six batches of human plasma for the analytes at low and high quality control concentrations. The precision and accuracy for atorvastatin at the LQC concentration were found to be 1.48% and 101%, and at the HQC level they were 1.02% and 99.6%, respectively. Similarly, the precision and accuracy for aspirin at the LQC concentration were found to be 3.30% and 99.8%, and at the HQC level they were 0.53% and 93.0%, respectively.

### Calibration curve and linearity

The ten–point calibration curve was found to be linear over the concentration range of 0.20–151 ng/mL for atorvastatin and 15.0–3000 ng/mL for aspirin. After comparing the two weighting models (1/x and 1/x^2^), a regression equation with a weighting factor of 1/x^2^ of the drug to the IS concentration was found to produce the best fit for the concentration–detector response relationship for both of the analytes in human plasma. The mean correlation coefficient of the weighted calibration curves generated during the validation was ≥ 0.99.

### Precision and accuracy

The results for intra–day and inter–day precision and accuracy in plasma quality control samples are summarized in Table 2. The intra–day and inter-day precision deviation values were all within 15% of the relative standard deviation (RSD) at the low, middle 1, middle 2, and high quality control levels, and also within 20% at the LLOQ QCs level. The intra–day and inter–day accuracy deviation values were all within 100 ± 15% of the actual values at low, middle 1, middle 2, and high quality control levels, and also within 100 ± 20% at LLOQ QCs level. The results revealed high precision and accuracy.

### Extraction efficiency

Simple liquid–liquid extraction with MTBE proved to be robust and provided the cleanest samples. The recovery of analytes and internal standards were high and reproducible. The mean overall recovery results are summarized in Table 3.

### Dilution integrity

The upper concentration limits can be extended to 241 ng/mL for atorvastatin and 4800 ng/mL for aspirin by 2–fold and 4–fold dilutions with screened blank human plasma. The mean back-calculated concentrations for the 2–fold and 4–fold dilution samples were within 85–115% of their nominal values. The coefficients of variation (%CV) for the 2–fold and 4–fold dilution samples were less than 10%.

### Stability studies

In the different stability experiments carried out viz. benchtop stability (8 h), autosampler stability (48 h), repeated freeze–thaw cycles (4 cycles), reinjection stability (24 h), wet extract stability (24 h at 2–8 °C), and long term stability at −70 °C for 50 days, the mean % nominal values of the analytes were found to be within ±15% of the predicted concentrations for the analytes at their LQC and HQC levels (Table 4). Thus, the results were found to be within the acceptable limits during the entire validation.

### Pharmacokinetic study results

**Fig. 4.**
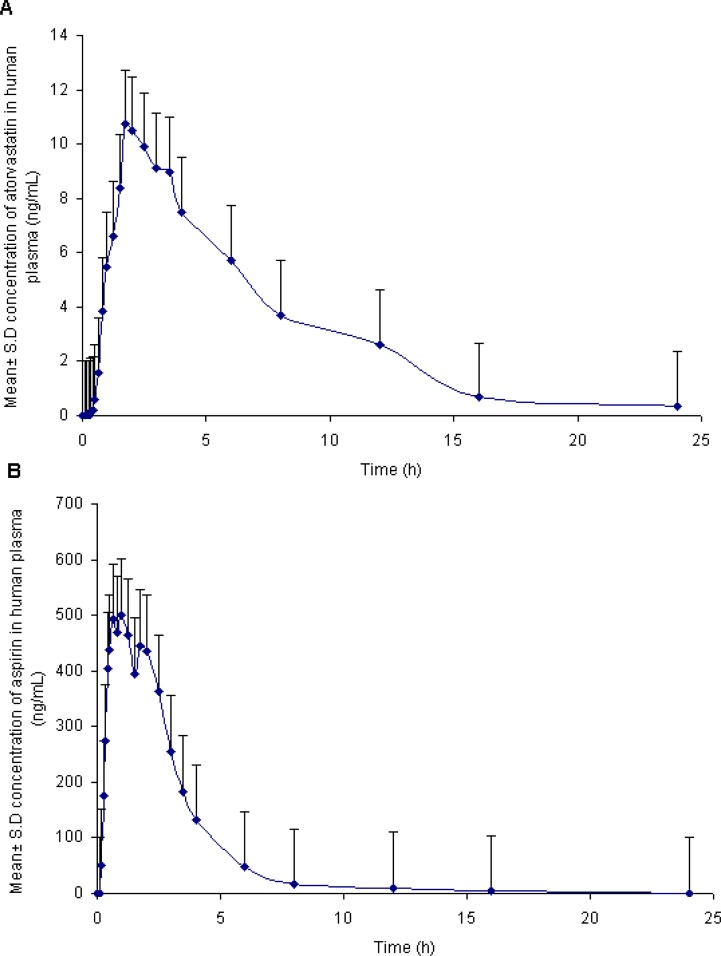
Mean plasma concentration–time profile of atorvastatin (A) and aspirin (B), in human plasma following oral dosing of atorvastatin (20 mg) and aspirin (75 mg) tablet to healthy volunteers (n = 6).

In order to verify the sensitivity and selectivity of this method in a real–time situation, the present method was used to analyze the atorvastatin and aspirin concentrations in human plasma samples collected from healthy male volunteers (n = 6). The mean plasma concentration vs. time profiles for atorvastatin and aspirin are shown in Figure 4, and the corresponding pharmacokinetic parameters are listed in Table 5. The pharmacokinetic parameters of the atorvastatin compares with the values reported earlier [17]. The terminal half-life (*t*_1/2_) was marginally lower than the reported values while the AUC, *C*_max,_ and *t*_max_ values were somewhat higher. This may be due to the differences in genetics, race, age, and gender (body size and muscle mass) of the study subjects or to the differences in the type of food consumed. Whereas the pharmacokinetic values of aspirin were in close proximity to the values reported by earlier researchers [30].

### Incurred sample reanalysis

Since then, the FDA has introduced the necessity of the incurred sample reanalysis evaluation at the Crystal City III meeting [31], stating that it is necessary to demonstrate assay reproducibility by using dosed subject samples. Incurred sample reanalysis (ISR) was performed using two plasma samples from each subject and re–assayed in a separate batch run. The differences in concentrations between the ISR and the initial values for all of the tested samples were less than 12% (Table 6), indicating good reproducibility of the present method.

## Conclusions

The LC–MS/MS assay method described in this paper is simple, rapid, specific, and sensitive for the quantification of atorvastatin and aspirin in human plasma using the polarity–switching mode, and is fully validated as per the FDA guidelines. To the best of our knowledge, this is the first report on the simultaneous assay of atorvastatin and aspirin in any of the matrices without compromising on the reported sensitivity for each analyte. The method was found to be suitable for pharmacokinetic studies in humans. The simple liquid–liquid extraction method gave consistent and reproducible recoveries for the analytes from plasma. The method provided good linearity. A sample turnover rate of less than 3.0 min makes it an attractive procedure in the high–throughput bioanalysis of atorvastatin and aspirin.

## Figures and Tables

**Tab. 1.** Tandem mass-spectrometer main working parameters.

**Parameter**	**Analyte**			
	**Atorvastatin**	**Proguanil**	**Aspirin**	**Furosemide**
Mode of analysis	Positive	Positive	Negative	Negative
Ion transition, m/z	559.2/440.0	254.2/170.1	179.0/136.6	329.2/285.0
Source temperature, °C	500	500	500	500
Dwell time per transition, msec	200	200	200	200
Nebulizer gas, psi	40	40	40	40
TurboIon gas, psi	35	35	35	35
Curtain gas, psi	20	20	20	20
Collision gas, psi	8	8	8	8
Ion spray voltage, V	5500	5500	−4500	−4500
Entrance potential, V	10	10	−10	−10
Declustering potential, V	100	60	−40	−60
Collision energy, V	30	20	−9	−25
Collision cell exit potential, V	12	9	−5	−6
Resolution	Unit	Unit	Unit	Unit

**Tab. 2.** Precision and accuracy data for atorvastatin and aspirin in human plasma samples.

**Analyte**	**Concentration added (ng/mL)**	**Intra-day precision and accuracy (*n* =12; 6 from each batch)**		
		**Concentration found (mean ± S.D; ng/mL)**	**Precision (%)**	**Accuracy (%)**

Atorvastatin	0.20	0.20 ± 0.01	4.82	100
"	0.61	0.61 ± 0.01	1.50	100
"	22.3	22.2 ± 0.12	0.54	100
"	75.4	75.4 ± 0.85	1.12	99.9
"	130	130 ± 0.73	0.56	99.9
Aspirin	15.7	14.8 ± 0.91	6.15	93.8
"	45.0	45.1 ± 2.09	4.63	100
"	450	457 ± 15.1	3.29	102
"	1500	1491 ± 54.5	3.65	99.4
"	2500	2513 ± 124	4.94	101

**Analyte**	**Concentration added (ng/mL)**	**Inter-day precision and accuracy (*n* =30; 6 from each batch)**		
		**Concentration found (mean ± S.D; ng/mL)**	**Precision (%)**	**Accuracy (%)**

Atorvastatin	0.20	0.20 ± 0.01	4.36	101
"	0.61	0.61 ± 0.01	3.76	99.9
"	22.3	22.0 ± 0.74	3.39	98.8
"	75.4	75.1 ± 1.00	1.33	99.5
"	130	130 ± 1.08	0.83	99.9
Aspirin	15.7	15.1 ± 1.03	6.82	95.6
"	45.0	44.9 ± 2.00	4.45	99.8
"	450	455 ± 12.3	2.71	101
"	1500	1488 ± 69.5	4.67	99.2
"	2500	2478 ± 122	4.92	99.1

**Tab. 3.** Mean overall recoveries of atorvastatin, aspirin, proguanil and furosemide (*n*=6).

**Analyte**	**Sample concentr. (ng/mL)**	**Response unextracted (mean ± SD)**	**Response extracted (mean ± SD)**	**Recovery (%)**	**Mean ± SD (% CV) recovery**
Atorvastatin	0.61	77796 ± 1615	95124 ± 2607	81.8	82.3 ± 0.61 (0.74%)
"	75.4	2657211 ± 119695	3233995 ± 140199	82.2	
"	130	4472209 ± 184439	5390016 ± 240360	83.0	

Aspirin	45.0	12669 ± 215	14618 ± 223	86.7	87.2 ± 0.50 (0.57%)
"	1500	434631 ± 16537	498632 ± 11609	87.2	
"	2500	668653 ± 15788	762781 ± 14279	87.7	

Proguanil	1000	104442 ± 4458	126682 ± 3255	82.4	–

Furosemide	1000	2321440 ± 72485	2768270 ± 151484	83.9	–

**Tab. 4.** Stability data for atorvastatin and aspirin in human plasma samples (*n*=6).

**Stability test**	**Atorvastatin**			
	**QC (spiked concentr., ng/mL)**	**Mean±SD (ng/mL)**	**Accuracy/Stability (%)**	**Precision (%)**

Aautosampler stability (at 10 °C for 48 h)	0.61	0.60 ± 0.02	97.9	2.88
	130	131 ± 2.21	101	1.68
Wet extract stability (at 2–8 °C for 24 h)	0.61	0.60 ± 0.02	98.1	2.65
	130	130 ± 1.99	99.9	1.53
Bench top stability (8 h at room temp.)	0.61	0.59 ± 0.02	96.5	2.91
	130	129 ± 1.89	99.2	1.47
Freeze-thaw stability (4 cycles)	0.61	0.60 ± 0.04	97.8	6.89
	130	134 ± 3.19	103	2.37
Reinjection stability (24 h)	0.61	0.60 ± 0.01	98.8	2.53
	130	124 ± 2.17	98.2	1.31
Long-term stability (at −70 °C for 50 days)	0.61	0.60 ± 0.02	99.9	1.54
	130	130 ± 1.59	100	2.12

**Stability test**	**Aspirin**			
	**QC (spiked concentr., ng/mL)**	**Mean±SD (ng/mL)**	**Accuracy/Stability (%)**	**Precision (%)**

Aautosampler stability (at 10 °C for 48 h)	45.0	44.9 ± 0.90	99.8	2.00
	2500	2687 ± 35.2	107	1.31
Wet extract stability (at 2–8 °C for 24 h)	45.0	44.9 ± 0.30	99.8	0.67
	2500	2641 ± 33.7	106	1.28
Bench top stability (8 h at room temp.)	45.0	44.4 ± 0.65	98.6	1.48
	2500	2519 ± 12.1	101	0.48
Freeze-thaw stability (4 cycles)	45.0	43.8 ± 0.80	97.3	1.82
	2500	2657 ± 23.5	106	0.89
Reinjection stability (24 h)	45.0	43.0 ± 060	98.6	1.39
	2500	2244 ± 22.7	92.7	1.01
Long-term stability (at −70 °C for 50 days)	45.0	44.5 ± 0.26	97.9	1.12
	2500	2565 ± 14.5	97.5	2.50

**Tab. 5.** Pharmacokinetic parameters of atorvastatin (20 mg) and aspirin (75 mg) (n=6, Mean±SD).

**Parameter**	**Atorvastatin**	**Aspirin**
*C*_max_ (ng/mL)	11.4 ± 1.57	552 ± 85.3
*t*_max_ (h)	2.2 ± 0.70	0.95 ± 0.60
AUC_0–t_ (ng h/mL)	74.1 ± 20.2	1632 ± 148
AUC_0–inf_ (ng h/mL)	76.7 ± 21.5	1655 ± 161
*t*_1/2_ (h)	4.1 ± 1.03	3.07 ± 1.99
*Ke* (h^−1^)	0.18 ± 0.06	0.31 ± 0.16
V_d_ (L)	1.55 ± 0.15	0.20 ± 0.12
CL (L/h/kg)	0.28 ± 0.09	0.05 ± 0.00

**Tab. 6.** Incurred samples re-analysis data of atorvastatin and aspirin.

**Sample**	**Atorvastatin**			**Aspirin**		

	**Initial conc. (ng/mL)**	**Re-assay conc. (ng/mL)**	**Difference^a^ (%)**	**Initial conc. (ng/mL)**	**Re-assay conc. (ng/mL)**	**Difference^a^ (%)**
1	13.4	13.0	−3.1	445	431	−3.3
2	8.4	7.9	−6.9	26.2	25.6	−2.0
3	7.7	6.9	−10.4	520	555	6.4
4	9.2	10.4	11.8	44.4	40.6	−9.0
5	8.7	9.0	3.3	326	337	3.3
6	6.0	6.3	5.6	116	127	8.8
7	8.9	9.0	1.2	386	401	3.9
8	1.1	1.0	−7.8	23.2	20.7	−11.5
9	10.8	11.6	7.1	327	345	5.2
10	3.5	3.8	7.5	26.6	25.3	−4.9
11	8.5	9.5	11.1	378	362	−4.3
12	4.1	4.3	4.0	22.5	24.6	9.1

a Expressed as [(re-assay conc. − initial conc.)/average] × 100%.
